# Alcohol and tobacco dependence among medical practitioners

**DOI:** 10.1192/j.eurpsy.2024.829

**Published:** 2024-08-27

**Authors:** A. Belkahla, D. Brahim, I. Yaich, C. Ben Said, A. Ghenim, M. Mersni, H. Ben Said, N. Mechergui, I. Youssef, G. Bahri, N. Bram, N. Ladhari

**Affiliations:** ^1^Occupational pathology and fitness for work, Charles Nicolle Hospital, Tunis; ^2^Forensic Psychiatry department, Razi Hospital, Mannouba, Tunisia

## Abstract

**Introduction:**

Excessive use of tobacco, alcohol and other illicit drugs has a negative impact on the physical and mental health and work capacity of users. Physicians are no exception to these dreadful practices.

**Objectives:**

To assess tobacco and alcohol use among medical staff and the factors associated with these uses.

**Methods:**

Descriptive cross-sectional study of physicians practicing in different Tunisian hospitals. The levels of tobacco and alcohol dependence were assessed by the Fangeström and AUDIT tests. Anxiety and depression disorders were screened by the hospital anxiety and depression scale (HAD)

**Results:**

A total of 45 physicians participated in our study. The average professional seniority was 3.36 ± 3.5 years. The mean age was 32.11 ± 6.08 years with a sex ratio (M/F) of 0.32. The participants were medical residents in 64% of the cases. The frequency of smoking was estimated at 24%. The level of smoking dependence was high in 9% of cases. Men were more addicted to nicotine than women (p=0.014). Alcohol consumption was 18%, made up of 62% of women; with a strong dependence rate in 25% of users. Definite anxiety disorders were found in 7% of cases and definite depressive disorders were present in 13% of cases. No correlation between medical specialty, grade, anxiety disorders and level of dependence was observed.

**Conclusions:**

Doctors seem to be particularly affected by addictive behaviours and psycho-emotional disorders which could sustain these practices. Awareness-raising sessions and special monitoring must be introduced to combat these scourges.

**Disclosure of Interest:**

None Declared

